# Knockout of vasohibin‐2 reduces tubulin carboxypeptidase activity and increases paclitaxel sensitivity in ovarian cancer

**DOI:** 10.1002/cam4.3841

**Published:** 2021-03-12

**Authors:** Takahiro Koyanagi, Yasushi Saga, Yoshifumi Takahashi, Kohei Tamura, Takahiro Yoshiba, Suzuyo Takahashi, Akiyo Taneichi, Yuji Takei, Masashi Urabe, Hiroaki Mizukami, Hiroyuki Fujiwara

**Affiliations:** ^1^ Department of Obstetrics and Gynecology School of Medicine Jichi Medical University Shimotsuke Tochigi Japan; ^2^ Division of Genetic Therapeutics Center for Molecular Medicine Jichi Medical University Shimotsuke Tochigi Japan

**Keywords:** CRISPR/Cas9, detyrosinated tubulin, ovarian cancer, paclitaxel, vasohibin‐2

## Abstract

Vasohibin‐1 (VASH1) is a VEGF‐inducible endothelium‐derived angiogenesis inhibitor, and vasohibin‐2 (VASH2), its homolog, exhibits proangiogenic activity. VASH2 is expressed by various cancer cells and accelerates tumor angiogenesis and progression. VASH2 was recently shown to exhibit tubulin carboxypeptidase (TCP) activity related to microtubule functions. Paclitaxel (PTX), an effective chemotherapeutic agent that is widely used to treat ovarian cancer, inhibits microtubule depolymerization and may interact with VASH2. We herein established several VASH2 knockout ovarian cancer cell lines using the CRISPR/Cas9 genome editing system to examine the intracellular tubulin detyrosination status and PTX chemosensitivity. The knockout of VASH2 did not affect the proliferation or sphere‐forming activity of ovarian cancer cells in vitro. A Western blot analysis of VASH2 knockout cells revealed the weak expression of detyrosinated tubulin and upregulated expression of cyclin B1. The knockout of VASH2 significantly increased chemosensitivity to PTX, but not to cisplatin in ovarian cancer cell lines. The knockout of VASH2 reduced TCP activity and increased cyclin B1 expression, resulting in increased PTX chemosensitivity in ovarian cancer cells. The inhibition of angiogenesis and regulation of microtubule activity may be achieved in ovarian cancer treatment strategies targeting VASH2.

## Graphical abstract

In the present study, we demonstrated that the knockout of the angiogenesis stimulator, vasohibin‐2 (VASH2) reduced tubulin carboxypeptidase (TCP) activity, increased cyclin B1 expression, and increased paclitaxel chemosensitivity in ovarian cancer cells. The inhibition of angiogenesis and regulation of microtubule activity may be expected in ovarian cancer treatment strategies targeting VASH2.

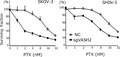

## 1 INTRODUCTION

In industrialized countries, the second most common gynecological malignancy and leading cause of cancer death is ovarian cancer.^1^ The standard treatments for ovarian cancer are debulking surgery and adjuvant chemotherapy with platinum and taxane, with approximately 80% of patients initially achieving complete responses. However, abdominal recurrence with reduced chemosensitivity is common, ultimately leading to death due to disease progression.^2^, ^3^ A large number of clinical trials, including molecular targeted therapy, have investigated ovarian cancer treatments in an attempt to improve therapeutic outcomes; however, the findings obtained have not been satisfactory.^4^ Therefore, novel therapeutic strategies need to be developed.

The vasohibin family includes vasohibin‐1 (VASH1) and vasohibin‐2 (VASH2). VASH1 is an endothelial cell (EC)‐derived angiogenesis inhibitor, while VASH2 is a homolog of VASH1 that functions as a stimulator of angiogenesis.^5^, ^6^ VASH2 is produced by various cancer cells, including ovarian cancer, and promotes tumor growth by accelerating angiogenesis. VASH2 is not expressed by the majority of normal adult tissues, while VASH2 knockout mice are not embryonic lethal and have no major anomalies. Thus, the targeting of VASH2 may offer safer and more tumor‐specific favorable outcomes.^6^

Recent studies reported that VASH2 exhibited tubulin carboxypeptidase (TCP) activity related to microtubule polymerization.^7^, ^8^ Paclitaxel (PTX), an effective chemotherapeutic agent that is widely used to treat ovarian cancer, inhibits microtubule depolymerization and may interact with VASH2. However, the relationship between the TCP activity of VASH2 and cancer progression or drug sensitivity currently remains unclear.

CRISPR/Cas9 is a simple, but powerful tool that allows parts of the genome to be edited and has been increasingly performed in research in recent years. Double‐stranded breaks (DSBs) are induced at target DNA loci through the combined effects of a single guide RNA (sgRNA), which recognizes a specific DNA sequence, and the Cas9 nuclease.^9^ Non‑homologous end joining repairs DSBs, but frequently results in gene mutations, such as base insertions and deletions, which, if present in the coding region of the target gene, may knockout the expression of specific genes.^10^ Thus, the specific knockout of VASH2 may be induced in ovarian cancer cells using CRISPR/Cas9.

Therefore, we herein established several VASH2 knockout ovarian cancer cell lines using the CRISPR/Cas9 genome editing system to examine the intracellular tubulin detyrosination status and PTX chemosensitivity.

## 2 MATERIALS AND METHODS

### 2.1 Cell lines and culture

The human ovarian cancer cell line SKOV‐3 was purchased from the American Type Culture Collection (Manassas, VA, USA). The human ovarian serous adenocarcinoma cell line SHIN‐3 was provided by Dr. Y. Kiyozuka (Kurume University of Medicine, Japan). Dulbecco's Modified Eagle Medium/F12 (DMEM/F12; Thermo Fisher Scientific, Inc., Waltham, MA, USA) containing 10% of inactivated fetal bovine serum (FBS; Sigma‐Aldrich; Merck KGaA, Darmstadt, Germany) and 1% of penicillin/streptomycin (Thermo Fisher Scientific, Inc.) was used as the basal medium to culture these cells at 37°C in a humidified atmosphere under 5% CO_2_.

### 2.2 Construction of the plasmid vector

The Cas9 expression vector (pCMV‐Cas9‐HA‐IRES‐bsr) was used as described previously.^11^ The target VASH2 sequence for CRISPR was searched for by Optimized CRISPR Design (crispr.mit.edu/), and that with the highest score was used. A vector expressing an sgRNA targeting VASH2 (sgVASH2) (pRGEN‐U6‐sgVASH2) was produced by annealing two DNA oligonucleotides, 5′‐ CACCGAATGGCCGCTATGGCTCATT‐3′ and 5′‐ AAACAATGAGCCATAGCGGCCATTC‐3′, and inserting them at the BsaI site of pRGEN‐U6‐sgRNA (Toolgen).

### 2.3 Establishment of VASH2 knockout ovarian cancer cell lines

The co‐transfection of SKOV‐3 and SHIN‐3 cells with pCMV‐Cas9‐HA‐IRES‐bsr and pRGEN‐U6‐sgRNA or pRGEN‐U6‐sgVASH2 was conducted using Lipofectamine LTX and Plus Reagent (Thermo Fisher Scientific, Inc.). After culturing on cell culture media supplemented with 10 μg/ml of blasticidin S hydrochloride (Funakoshi, Tokyo, Japan) to obtain single colonies, transfected cells were selected.

### 2.4 Detection of mutations in the VASH2 genome

pCMV‐Cas9‐HA‐IRES‐bsr‐ and pRGEN‐U6‐sgVASH2‐transfected cells were seeded at a density of 2 × 10^5^ cells/well on 6‐well plates, harvested after 24 hours using trypsin, and the extraction of DNA was then performed using the QIAamp^®^ DNA Mini kit (Qiagen GmbH, Hilden, Germany). In order to amplify the VASH2 genome, extracted DNA was used as the template for PCR with the TaKaRa Ex Taq Hot Start version (Takara Bio Inc.) and PTC‐100 (Bio‐Rad Laboratories, Inc., Hercules, CA, USA). The primers used in the reaction were as follows: Forward: 5′‐CTACTTAACCAATGGGCAGC‐3′, reverse: 5′‐CTTCATTCTCATGTCCCTGG‐3′. PCR involved 40 cycles of heating at 95°C for 30 seconds (denaturation), at 56°C for 30 seconds (annealing), and at 75°C for 30 seconds (extension). The PCR product was cloned using the Mighty TA‐cloning kit (Takara Bio Inc.), and Sanger sequencing was performed with the Applied Biosystems 3730xl DNA Analyzer (Thermo Fisher Scientific, Inc.).

### 2.5 Reverse transcription‐polymerase chain reaction (RT‐PCR)

Total RNA was extracted from cell cultures with ISOGEN II (Nippon Gene, Toyama, Japan) according to the manufacturer's instructions. The concentration of extracted RNA was assessed using the NanoDrop 2000c spectrophotometer (Thermo Scientific, Wilmington, DE). The RT‐PCR procedure was performed with the reagent SuperScript^TM^ III One‐Step RT‐PCR System with Platinum^TM^ *Taq* DNA Polymerase (Invitrogen) in the Veriti Thermal Cycler (Thermo Fisher Scientific, Inc.). cDNA synthesis was achieved in a 30‐minute incubation at 55℃. The following PCR conditions were then used: an initial denaturation step at 94°C for 2 minutes, followed by 35 cycles at 94°C for 30 seconds (denaturation), at 56°C for 30 seconds (annealing), and at 72°C for 30 seconds (extension). A 2% of agarose gel was used to separate PCR products, which were visualized under ultraviolet light by ethidium bromide staining. The following primer pairs were used: the human glyceraldehyde‐3‐phosphate dehydrogenase gene (GAPDH) forward primer, 5′‐ACCACAGTCCATGCCATCAC‐3′, and reverse primer, 5′‐TCCACCACCCTGTTGCTGTA‐3′; and the human VASH2 forward primer, 5′‐ CTAAGGGGGGAGAAATGGTG‐3′, and reverse primer, 5′‐ TTCTCACTTGGGTCGGAGAG ‐3′.

### 2.6 Cell growth curve

Tumor cells were seeded at a density of 500 cells/well on 96‐well plates, and 10 μL of the Premix WST‐1 Cell Proliferation Assay System (Takara Bio Inc., Tokyo, Japan) was applied daily to each well. Twenty‐four hours after the application of Premix, absorption was measured at 450 nm using SpectraMax 190 (Molecular Devices, LLC, Sunnyvale, CA, USA), and a cell growth curve was prepared.

### 2.7 Three‐dimensional (3D) culture

Tumor cells were seeded at a density of 1 × 10^4^ cells/well on a 6‐well plate and then cultured in DMEM/F12 with 1% of low melting temperature agarose (FMC Bioproducts, Rockland, ME, USA) containing with 10% of FBS. The diameters of acini were measured 7 days later under an inverted microscope (IX73l; Olympus Corporation, Tokyo, Japan).

### 2.8 Western blot analysis

Tumor cells were seeded at a density of 2 × 10^5^ cells/well on 6‐well plates. After a 24‐h incubation, cells were exposed to 10 nM of PTX for 1 hour and then lysed with cell lysis buffer (1% NP‐40, 150 mM NaCl, and 50 mM Tris‐HCl, pH 8.0). The proteins extracted were mixed with 1% of sodium dodecyl sulfate (SDS) sample buffer (10 mM Tris‐HCl, pH 7.5, 150 mM NaCl, 1% SDS, and EDTA‐free Protease inhibitor cocktail) (Roche, Basel, Switzerland), electrophoretically separated on 10% of polyacrylamide gels, and transferred to polyvinylidene fluoride (PVDF) membranes (Merck KGaA, Darmstadt, Germany). These membranes were left to stand at room temperature for 1 hour in PVDF Blocking Reagent for Can Get Signal^®^ (Toyobo Life Science, Osaka, Japan), subjected to washing using Tris‐buffered saline‐Tween‐20 (TBS‐T) three times, and then, incubated at room temperature overnight with the following antibodies in Can Get Signal^®^ Immunoreaction Enhance Solution 1 (Toyobo Life Science): anti‐detyrosinated alpha tubulin rabbit polyclonal (cat. no. ab48389; Abcam, Cambridge, UK), anti‐alpha tubulin mouse monoclonal (cat. no. sc‐32293; Santa Cruz Biotechnology, Inc., Dallas, TX, USA), anti‐cyclin B1 rabbit monoclonal (cat. no. 12231; Cell Signaling Technology, Inc., Danvers, MA, USA), and anti‐actin rabbit polyclonal (cat. no. A2066; Sigma‐Aldrich; Merck KGaA) antibodies. Once the reaction was complete, the membranes were subjected to washing with TBS‐T three times, and then, incubated with the peroxidase‐labeled anti‐mouse or anti‐rabbit antibody (GE Healthcare Japan, Tokyo, Japan) in Can Get Signal^®^ Immunoreaction Enhance Solution 2 (Toyobo Life Science) at room temperature for 1 hour. Membranes were again washed with TBS‐T three times, incubated with ECL prime Western blotting detection reagent (GE Healthcare Japan), and imaged with the cooled CCD system (LAS‐4000mini: GE Healthcare Japan).

### 2.9 Colorimetric assay

The sensitivity of tumor cells to PTX (Bristol‐Myers Squibb Co., Ltd., Tokyo, Japan) and cisplatin (CDDP) (Bristol‐Myers Squibb Co., Ltd., Tokyo, Japan) was investigated by a colorimetric assay using the Premix WST‐1 Cell Proliferation Assay System (Takara Bio Inc., Tokyo, Japan). Tumor cells were exposed to each drug at concentrations of 1–32 nM (for PTX) or 1–32 μM (for CDDP) for 48 hours. The viable cell count measured by the colorimetric assay was presented as a percentage to the count of the control untreated with anticancer drugs. A dose‐response curve was prepared, and the 50% growth‐inhibitory concentration (IC_50_) was obtained for each anticancer agent.

### 2.10 Statistical analysis

Statistical analyses were performed using EZR (Saitama Medical Center, Jichi Medical University, Saitama, Japan). The significance of differences between two groups was assessed using the Student's *t* test. *p* < 0.05 was considered to be significant.

## 3 RESULTS

### 3.1 Establishment of VASH2 knockout ovarian cancer cell lines

**FIGURE 1:**
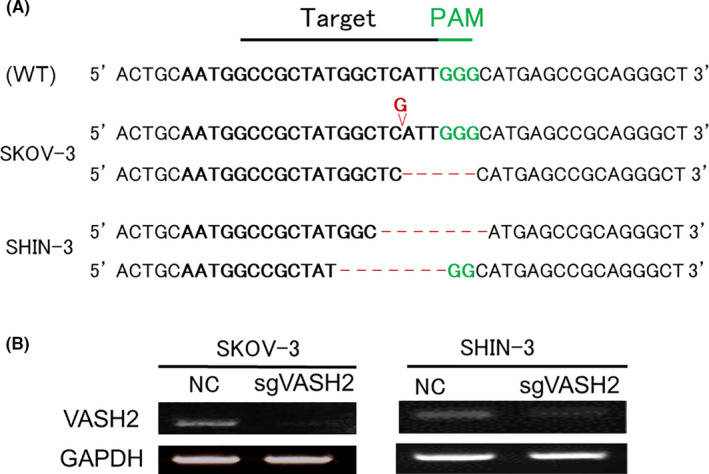
Confirmation of the knockout of the *VASH2* gene in ovarian cancer cell lines. (A) After transfection of the target CRISPR/Cas9 plasmid vectors, the *VASH2* gene was sequenced by Sanger sequencing. A 1‐base insertion and 6‐base deletion were identified around the targeted region in SKOV‐3‐transfected cells, and a 9‐base deletion and different 9‐base deletion around the targeted region in SHIN‐3‐transfected cells. (B) The efficient knockout of the *VASH2* gene was observed by RT‐PCR in each ovarian cancer cell line. The VASH2 knockout ovarian cancer cell lines SKOV‐3/sgVASH2 and SHIN‐3/sgVASH2 were established. WT, wild type; NC, negative control

The *VASH2* gene was subjected to Sanger sequencing to confirm the knockout of VASH2 in pCMV‐Cas9‐HA‐IRES‐bsr and pRGEN‐U6‐sgVASH2‐transfected cells. A 1‐base insertion and 6‐base deletion were identified around the targeted region in SKOV‐3‐transfected cells, while a 9‐base deletion and different 9‐base deletion were detected around the targeted region in SHIN‐3‐transfected cells (Figure 1A), confirming successful genome editing for VASH2 by CRISPR/Cas9. RT‐PCR also revealed the efficient knockout of the *VASH2* gene in each cell line (Figure 1B). The VASH2 knockout ovarian cancer cell lines SKOV‐3/sgVASH2 and SHIN‐3/sgVASH2 were established. pCMV‐Cas9‐HA‐IRES‐bsr and pRGEN‐U6‐sgRNA‐transfected cells were used as negative controls (SKOV‐3/NC and SHIN‐3/NC) in subsequent experiments.

### 3.2 Cell growth curve

**FIGURE 2:**
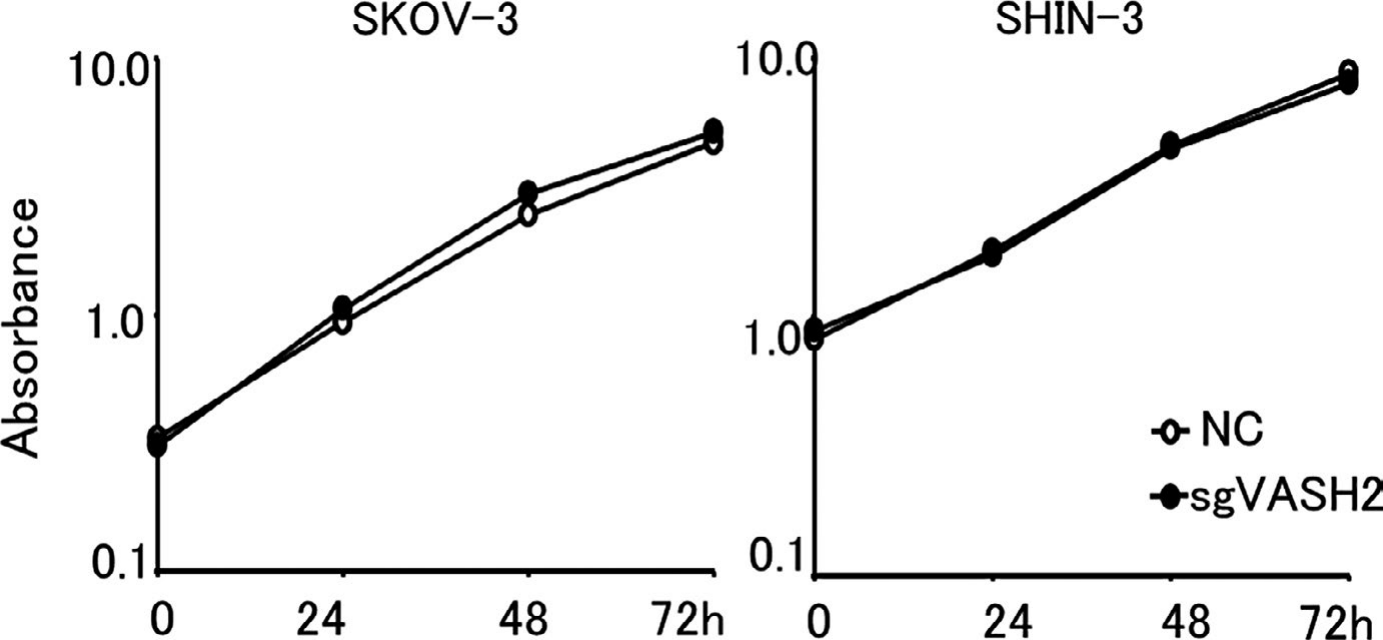
Effects on the in vitro proliferation of cancer cells. Line graphs show the proliferation of SKOV‐3 and SHIN‐3 cells 24, 48, and 72 h after seeding. The knockout of VASH2 had no impact on cell proliferation. Data are shown as means and SD (n = 3). NC, negative control

The effects of the VASH2 knockout on cell growth were evaluated based on comparisons of the growth of control and VASH2 knockout ovarian cancer cell lines in vitro. Significant differences were not noted in cell growth between negative control (NC) and VASH2 knockout cells (Figure 2), suggesting the lack of an effect of the VASH2 knockout on cell growth in vitro.

### 3.3 3D culture

**FIGURE 3:**
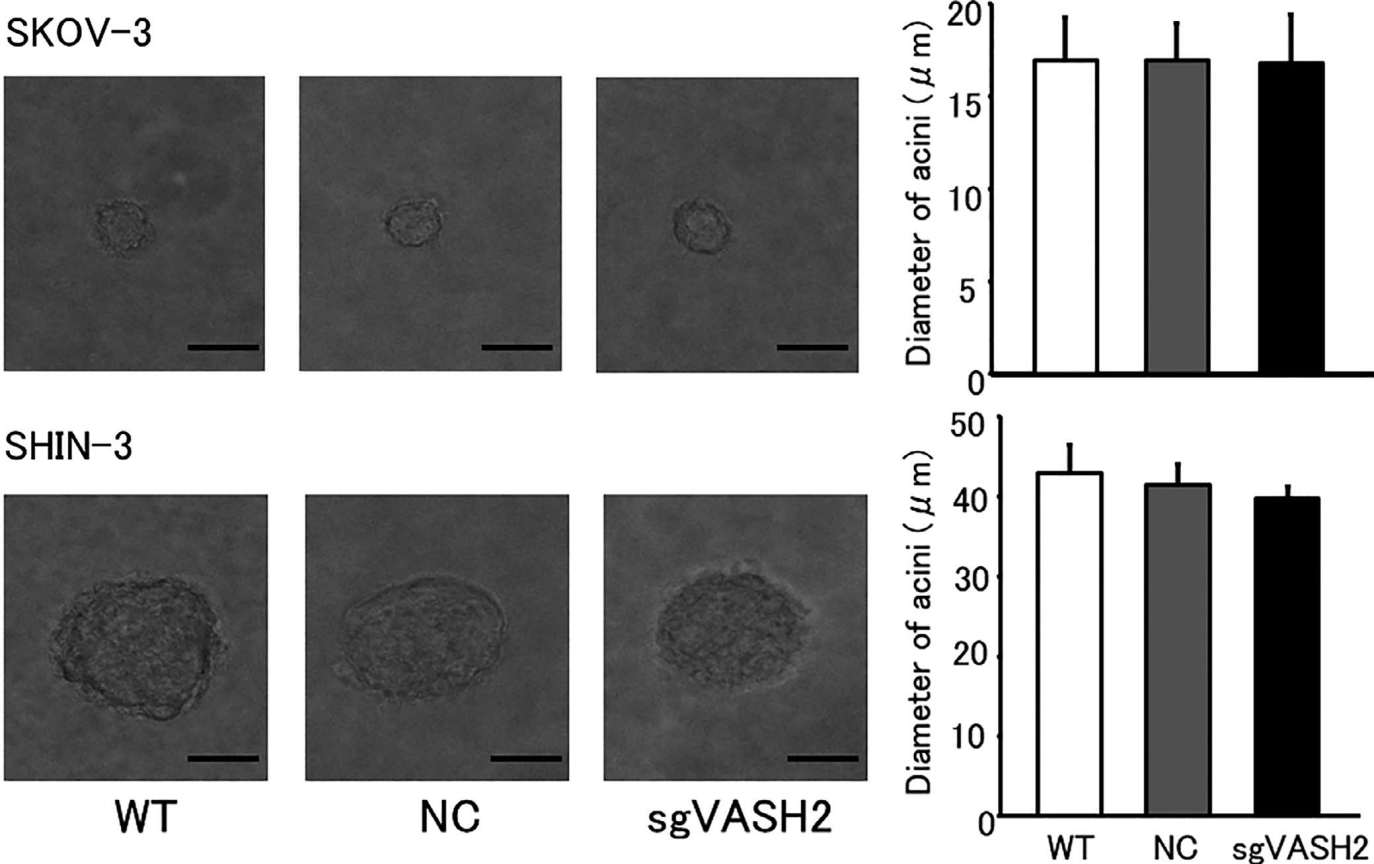
Microscopic figures of the 3D culture. Each colony was photographed under an inverted microscope 7 days after seeding. The bar graph shows the diameters of acini. The knockout of VASH2 had no impact on sphere‐forming activity in the 3D culture. Data are shown as means and SD (n = 3). WT, wild type; NC, negative control. Bar = 20 μm

Comparisons of cell growth in 3D cultures were performed between NC and the VASH2 knockout cells of each cell line. As shown in Figure 3, the diameters of the acini in either cell line did not significantly differ, demonstrating the lack of an effect of the knockout of VASH2 on SKOV‐3 or SHIN‐3 cell growth in the 3D culture.

### 3.4 Tubulin detyrosination

**FIGURE 4:**
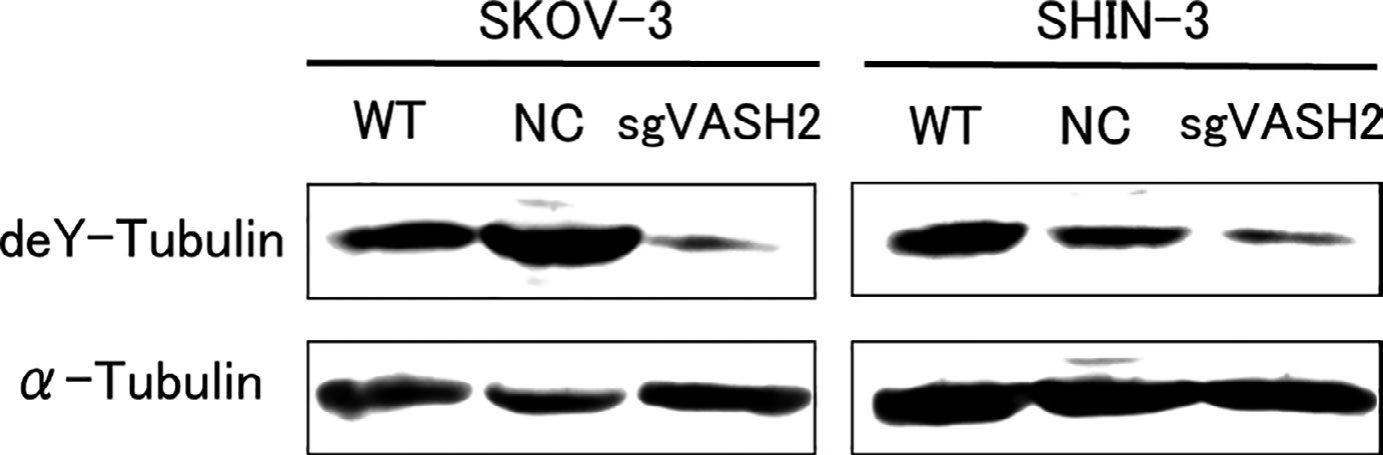
Western blotting of detyrosinated tubulin in VASH2 knockout or control ovarian cancer cell lines, SKOV‐3 and SHIN‐3. Parental and control cells clearly expressed detyrosinated tubulin in the presence of 10 nM PTX. In contrast, sgVASH2 cells weakly expressed detyrosinated tubulin. α‐Tubulin in the cell lysate was used as a loading control. WT, wild type; NC, negative control.

A Western blot analysis of VASH2 knockout or control ovarian cancer cell lines was performed and the results obtained are shown in Figure 4. Parental cells (WT) and NC both expressed detyrosinated tubulin in the presence of 10 nM PTX. In contrast, VASH2 knockout cells (sgVASH2) weakly expressed detyrosinated tubulin. These results suggested that the knockout of VASH2 inhibited tubulin detyrosination in ovarian cancer cells.

### 3.5 Cyclin B1 expression

**FIGURE 5:**
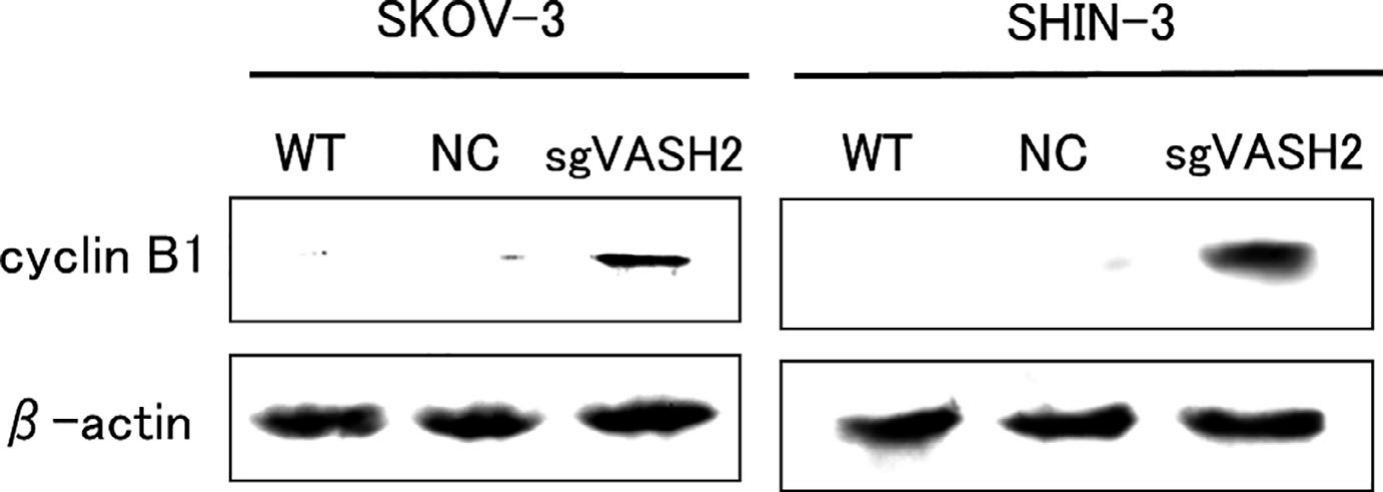
Western blotting of cyclin B1 in VASH2 knockout or control ovarian cancer cell lines, SKOV‐3 and SHIN‐3. Cyclin B1 expression was not detected in parental or control cells. However, sgVASH2 cells clearly expressed cyclin B1 at the position corresponding to a molecular weight of 55 kDa. β‐actin in the cell lysate was used as a loading control. WT, wild type; NC, negative control

Cyclin B1 expression was evaluated by a Western blot analysis. Its expression was not detected in WT or NC, whereas sgVASH2 cells clearly expressed cyclin B1 (Figure 5). These results suggest that the knockout of VASH2 increased cyclin B1 expression in ovarian cancer cells.

### 3.6 Chemosensitivity

**FIGURE 6:**
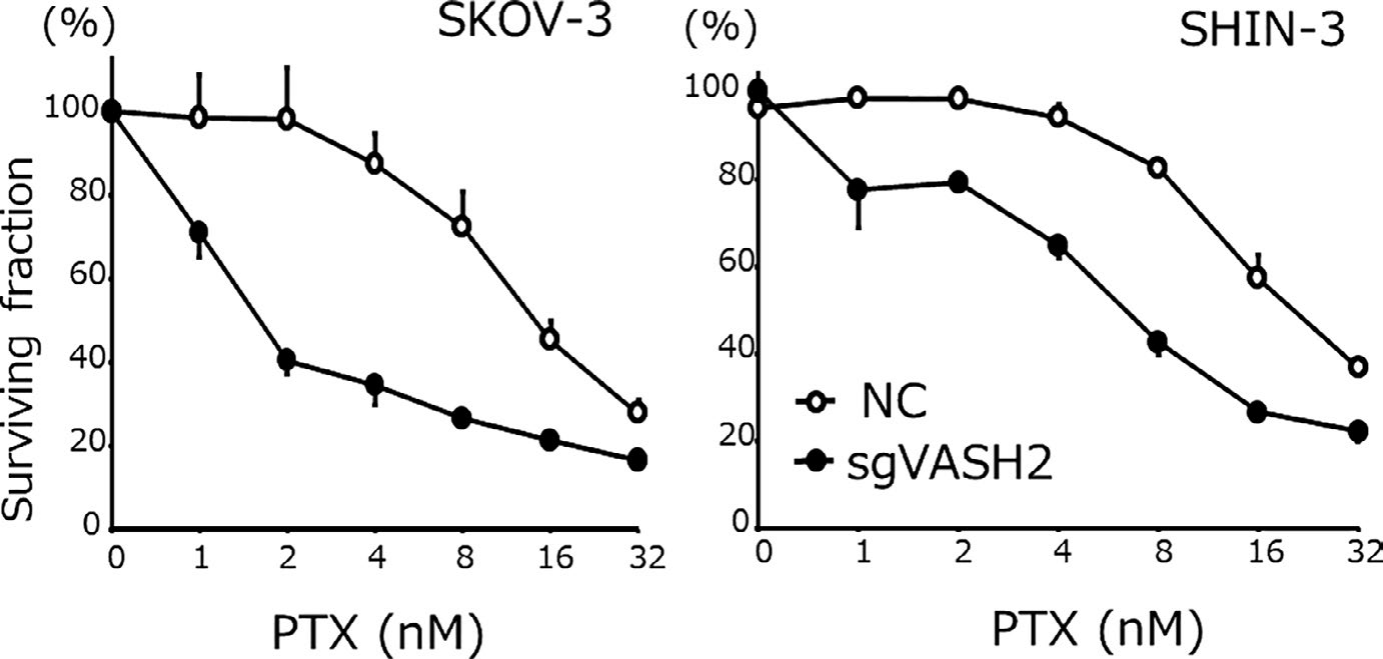
Chemosensitivity to PTX. The IC_50_ for PTX in SKOV‐3 were as follows: NC, 14.5 ± 1.3 nM vs. sgVASH2, 1.7 ± 0.1 nM (8.5‐fold higher sensitivity, *p* < 0.05). The IC_50_ for PTX in SHIN‐3 were as follows: NC, 21.4 ± 3.1 nM vs. sgVASH2, 6.7 ± 0.5 nM (3.2‐fold higher sensitivity, *p* < 0.05). Data are shown as means and SD (n = 3). PTX, paclitaxel; NC, negative control

**FIGURE 7:**
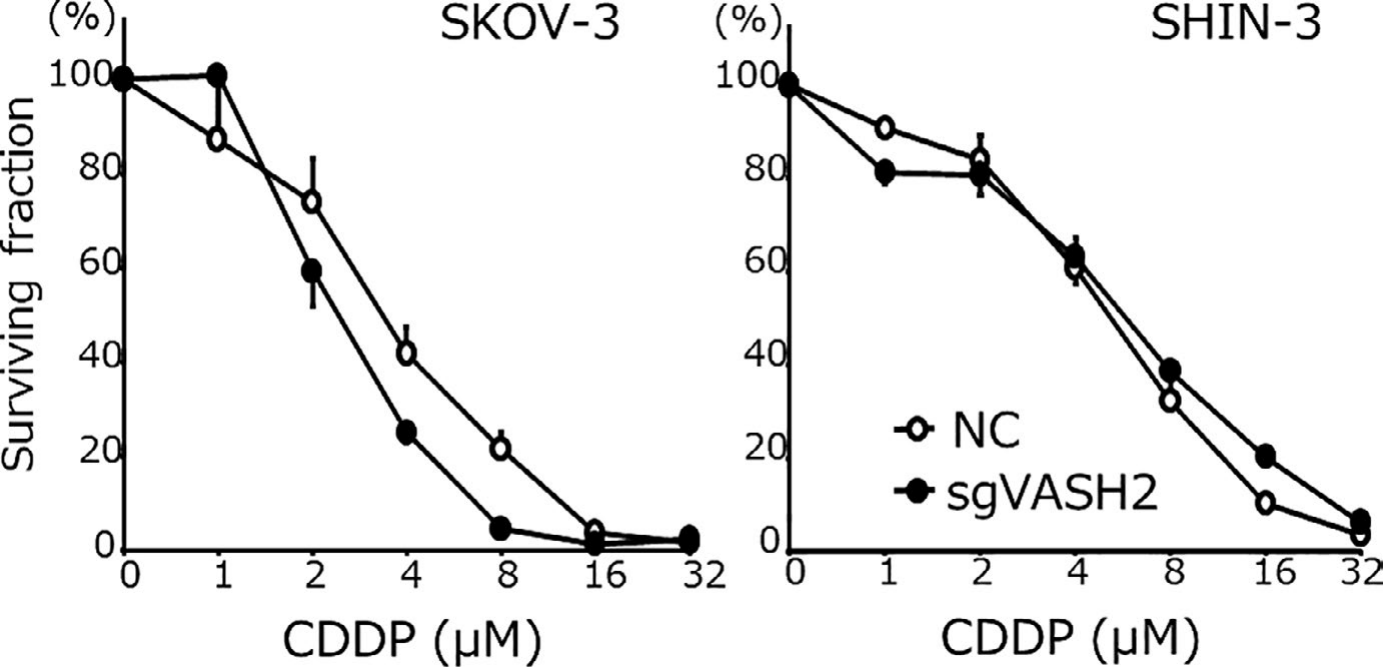
Chemosensitivity to CDDP. The IC_50_ for CDDP in SKOV‐3 were as follows: NC, 1.7 ± 0.2 μM vs. sgVASH2, 1.2 ± 0.2 μM (not significant). The IC_50_ for CDDP in SHIN‐3 were as follows: NC, 5.4 ± 0.7 μM vs. sgVASH2, 6.1 ± 0.4 μM (not significant). Data are shown as means and SD (n = 3). CDDP, cisplatin; NC, negative control

The sensitivity of each tumor cell line to PTX is shown in Figure 6. The IC_50_ for PTX in SKOV‐3/sgVASH2 was 1.7 ± 0.1 nM, which was 8.5‐fold higher than that in NC (14.5 ± 1.3 nM) (*p* < 0.05). Similarly, the IC_50_ for PTX in SHIN‐3/sgVASH2 was 6.7 ± 0.5 nM, which was 3.2‐fold higher than that in NC (21.4 ± 3.1 nM) (*p* < 0.05). In contrast, as shown in Figure 7, no significant difference was observed in the IC_50_ for CDDP in the SKOV‐3 clone; 1.2 ± 0.2 μM for sgVASH2 and 1.7 ± 0.2 μM for NC, respectively. Similarly, no significant difference was noted in the IC_50_ for CDDP in the SHIN‐3 clone; 6.1 ± 0.4 μM for sgVASH2 and 5.4 ± 0.7 μM for NC, respectively. Collectively, these results demonstrated that the knockout of VASH2 increased sensitivity to PTX, but not to CDDP in ovarian cancer cells.

## 4 DISCUSSION

In the present study, the knockout of VASH2 by CRISPR/Cas9 genome editing inhibited intracellular tubulin detyrosination and increased cyclin B1 expression. Furthermore, the knockout of VASH2 significantly increased sensitivity to PTX, but not to CDDP in ovarian cancer cell lines. These results suggest that VASH2 interacted with microtubules and may be a target for increasing the sensitivity of ovarian cancer cells to chemotherapy.

The expression of VASH2 has been detected in various cancer types and stimulates angiogenesis in a paracrine manner in neighboring vascular EC.^12^ Tumor angiogenesis was abrogated by the specific knockdown of intrinsic VASH2 in ovarian cancer cells, which inhibited tumor growth, peritoneal dissemination, and ascites production.^12^ Furthermore, the exogenous administration of siRNA targeting VASH2 with atelocollagen biomaterial to a murine xenograft model of ovarian cancer retarded tumor growth by suppressing angiogenesis.^13^ A previous study also showed that VASH2‐induced tumor angiogenesis and its antitumor activity were inhibited by a neutralizing anti‐VASH2 monoclonal antibody, similar to bevacizumab, without obvious side effects.^14^

Microtubules are a major component of the cytoskeleton, which comprises repeating α‐ and β‐tubulin heterodimers that are subjected to many posttranslational modifications, including the tyrosination–detyrosination cycle. Tubulin tyrosination is mediated by tubulin tyrosine ligase, and its detyrosination by TCP. The accumulation of detyrosinated tubulin has been reported in breast cancers and neuroblastomas with a poorer prognosis.^15^, ^16^ Vasohibin family members (VASH1 and VASH2) exhibit TCP activity, which catalyzes the C‐terminal tyrosine residue of α‐tubulin.^7^, ^8^ In the present study, VASH2 knockout cells weakly expressed detyrosinated tubulin, suggesting that VASH2 exhibits TCP activity and may play an important role in the accumulation of detyrosinated tubulin in ovarian cancer cells. However, the knockout of VASH2 did not completely inhibit tubulin detyrosination and had a negligible effect on the in vitro proliferation and sphere‐forming activity of ovarian cancer cells in 2D or 3D cell cultures, suggesting that microtubule functions are preserved. Therefore, factors other than the vasohibin family may be complementing TCP activity.

Another important function of microtubules is related to cell division. A complex of cyclin and cyclin‐dependent kinase has essential functions in cell cycle progression. Cyclin B1 is upregulated from the G2 to early M phase during the cell cycle. It subsequently forms a complex with cyclin‐dependent kinase 1 (CDK1), inducing the cell cycle to the middle M phase and decreasing its expression in the late M phase. PTX binds β‐tubulin and stabilizes microtubules by disrupting the dynamic equilibrium between soluble tubulin dimers and polymerized tubulin, resulting in an impaired metaphase to anaphase transition in the M phase, and ultimately cell apoptosis.^17^ Therefore, PTX exhibits strong antitumor activity against cancer cells, particularly in the middle M phase. The knockout of VASH2 induced the weaker expression of detyrosinated tubulin and stronger expression of cyclin B1, resulting in significantly increased PTX chemosensitivity, which may be attributed to the proportion of cells in the middle M phase being increased by the knockout of VASH2. It is speculated that the knockout of VASH2 inhibited the formation of spindle fibers that function in the late M phase, and, as a result, delayed the transition of the cell cycle from the middle M phase to the late M phase, leading to hypersensitivity to PTX. Consistent with the present results, the overexpression of cyclin B1 has been reported to sensitize cancer cells to PTX.^18^ On the contrary, the knockout of VASH2 did not affect sensitivity to CDDP in ovarian cancer cell lines, and this may be because CDDP is a drug that targets DNA itself, not microtubules.

Bevacizumab is a monoclonal antibody against vascular endothelial growth factor‐A (VEGF‐A) and has already been applied to various cancer treatments. Combination chemotherapy with PTX, carboplatin, and bevacizumab followed by maintenance treatment with bevacizumab is a standard regimen for advanced ovarian cancer. However, anti‐VEGF therapy causes adverse events, including drug resistance, toxic side effects, such as hypertension and proteinuria, and economic burden due to the prolonged treatment period. However, the targeting of VASH2 may prevent these events because of its function as a VEGF‐independent and EC‐extrinsic angiogenesis regulator that is weakly expressed by normal adult tissues, except the central nervous system and genital organs.^6^ Moreover, a haploinsufficient VEGF‐A gene was shown to cause embryonic lethality,^19^, ^20^ and its conditional knockout in EC resulted in multiple organ failure.^21^ In contrast to VEGF‐A knockout mice, VASH2 knockout mice are mostly viable, except for a minor abnormality in the placental vascular structure.^22^

VASH2 has multiple functions in tumor cells and the tumor microenvironment. It exhibits TCP activity intracellularly in tumor cells, and accelerates tumor angiogenesis in a paracrine manner in the tumor microenvironment. However, the relationship between the proangiogenic effects and TCP activity of VASH2 has not yet been elucidated in detail. Regarding the secretion of vasohibin family members, a specific protein called small vasohibin‐binding protein (SVBP) is essential for their efficient secretion.^23^ Further studies are needed to clarify the relationship between VASH2‐induced tubulin detyrosination and its secretion, including the SVBP expression status. Moreover, it currently remains unclear whether secreted VASH2 from cancer cells directly regulates tubulin detyrosination in ECs to promote their proliferation. Further studies that include in vivo experiments, such as combination therapy with an anti‐VASH2 monoclonal antibody and PTX, are needed under clinical settings.

In conclusion, the knockout of VASH2 reduced TCP activity, increased cyclin B1 expression, and increased PTX chemosensitivity in ovarian cancer cells. The inhibition of angiogenesis and regulation of microtubule activity may be expected with ovarian cancer treatment strategies targeting VASH2.

## CONFLICT OF INTEREST

The authors have no conflicts of interests.

## AUTHOR CONTRIBUTIONS

TK and YS performed experiments and wrote the manuscript. YT, KT, TY, ST, AT, YT, MU, and HM gave advice in preparing the manuscript. HF reviewed and edited the manuscript.

## Data Availability

The data that support the findings of this research are available from the corresponding author upon reasonable request.
